# Storage Proteins Are Driving Pediatric Hazelnut Allergy in a Lipid Transfer Protein-Rich Area

**DOI:** 10.3390/foods10102463

**Published:** 2021-10-15

**Authors:** Teresa Valbuena, Marta Reche, Guadalupe Marco, Inmaculada Toboso, Anna Ringauf, Israel J. Thuissard-Vasallo, Daniel Lozano-Ojalvo, Mónica Martínez-Blanco, Elena Molina

**Affiliations:** 1 Allergology Department, Hospital Universitario Infanta Sofía, San Sebastián de los Reyes, 28702 Madrid, Spain; marta.reche@salud.madrid.org (M.R.); guadalupe.marco@salud.madrid.org (G.M.); 2 Immunology, Laboratorio Central UR Salud Madrid, San Sebastián de los Reyes, 28702 Madrid, Spain; inmaculada.toboso@salud.madrid.org; 3 Macro Array Diagnostics, 1230 Wien, Austria; ringauf@macroarraydx.com; 4 Faculty of Biomedical and Health Science, Universidad Europea de Madrid, 28670 Madrid, Spain; israeljohn.thuissard@universidadeuropea.es; 5 Icahn School of Medicine at Mount Sinai, New York, NY 10029, USA; daniel.lozano-ojalvo@mssm.edu; 6 Instituto de Investigación en Ciencias de la Alimentación (CIAL) (CSIC-UAM), 28049 Madrid, Spain; m.martinez.blanco@csic.es (M.M.-B.); e.molina@csic.es (E.M.)

**Keywords:** hazelnut allergy, component-resolved diagnosis, skin-prick test, specific IgE, food challenge, severity

## Abstract

Oral food challenge (OFC) remains the gold standard for the diagnosis of food allergies. However, this test is not without risks, given that severe allergic reactions can be triggered while it is conducted. The purpose of this study is to identify potential demographic variables, clinical characteristics of the patients and biomarkers that may be associated with severe reactions during the hazelnut oral challenge test. The sample included 22 children allergic to hazelnut who underwent a tree nut skin prick test (SPT), specific IgE (sIgE) to hazelnut, component-resolved diagnosis (CRD) with different hazelnut allergens (Cor a 1, Cor a 8, Cor a 9, Cor a 11, Cor a 14), and a single-blind placebo-controlled challenge with hazelnut. A statistically significant relationship was found between the severity of the reaction and the highest values of sIgE to hazelnut, Cor a 11 and Cor a 14, cumulative symptom-triggering dose and sunflower seed sensitization. The use of the CRD is a useful tool to identify patients at higher risk of developing a severe reaction. In this pediatric population sample from Spain, storage proteins were confirmed to be most involved in hazelnut allergy and the development of severe reactions.

## 1. Introduction

Tree nut allergies are one of the most common allergies in pediatric patients. Hazelnut is one of the most common ones, especially in Europe. The frequency and clinical presentation of this allergy depend on the patient’s age and geographical location since they are influenced both by dietary habits in the patient’s location and by exposure to different kinds of pollen [1].

Allergy to hazelnut is one of the causes of anaphylaxis, and these severe reactions may occur even with very small amounts or when present as an allergen hidden in processed foods [2]. Additionally, allergy to hazelnut and tree nuts in general is often persistent throughout a patient’s life, resolving only in a limited number of patients (9–14%) [3].

Allergy to hazelnut is diagnosed based on a thorough medical history, the interpretation of SPT, and in vitro tests (sIgE to whole hazelnut extract or specific allergens). On many occasions, these diagnostic tools are not sufficient to reach a definitive diagnosis and an oral food challenge is required. This test remains the gold standard for food allergy diagnosis [4], but carries a high risk of unpredictable severe allergic reactions while it is conducted.

Being able to effectively establish a profile of the patients and identifying the biomarkers that may identify those at higher risk of developing a severe reaction during the oral challenge test would greatly improve food allergy management, thus avoiding unnecessary tests, which may entail a risk for the patient.

Some biomarkers analyzed are food SPT, sIgE to whole extract and sIgE to individual allergens. The dose of the allergen to which the patient is exposed is another factor to be considered. A priori, it could be expected that a higher allergen dose would result in more severe symptoms [5,6]; however, there are conflicting studies on the matter [7,8].

The food sensitization level, based both on the SPT and the sIgE result, as well as its association with the severity of the reaction is contradictory. Some studies have found a significant relationship between these biomarkers [5,8,9,10], while others have failed to establish this relationship [11,12,13,14,15].

Differences among the sensitization profiles found in patients can help us to recognize patients who can develop severe allergic reactions [16]. We can find studies in the literature associating positivity to some hazelnut storage proteins, such as Cor a 9 (11 S globulin) and Cor a 14 (2 S albumin) with severe reactions [17,18].

Thus, the purpose of this study is to identify potential demographical variables, patient clinical characteristics, and biomarkers which may be associated with severe reactions during hazelnut oral challenge tests in a sample of pediatric patients from a given geographical population.

## 2. Materials and Methods

### 2.1. Study Population

The study analyzed patients allergic to hazelnut included in a previous trial on epicutaneous immunotherapy with hazelnut (unpublished data), for which enrollment began in 2017 in the Allergology Department of Hospital Universitario Infanta Sofía, in San Sebastián de los Reyes (Madrid, Spain). The study enrolled pediatric patients younger than 16 with hazelnut allergic sensitization documented by means of a skin test and/or specific IgE and proven allergy to hazelnut based on a positive hazelnut challenge test.

Patient history was obtained for all patients, focusing on their allergies to tree nuts and, specifically, to hazelnut. Demographical variables and personal history of atopy, such as atopic dermatitis, rhinoconjunctivitis and bronchial asthma were collected. Allergy to other foods was diagnosed by compatible history together with proven food sensitization (positive SPT or sIgE) and in some patients by OFC.

The project was assessed and accepted by the Ethics Committee of Hospital Universitario La Paz (HULP Code: PI-2419). Before participating in the study, all parents and legal guardians of the patients enrolled signed a written informed consent.

### 2.2. Oral Hazelnut Challenges

The procedure was conducted based on the recommendations of the European Academy of Allergy and Clinical Immunology (EAACI) [19] and the PRACTALL consensus [20] on food challenge tests.

All patients underwent a single-blind placebo-controlled hazelnut oral challenge test. For each oral challenge test, the active agent and the placebo were administered on different, non-consecutive days.

All oral challenge tests were conducted under close monitoring and supervision of an allergologist and with qualified trained nurses [21].

The cumulative dose of hazelnut protein triggering symptoms was calculated in the result of the challenge. Hazelnut protein doses were given progressively and incrementally every 20 min, on the day of the active agent: 0.15 mg, 0.75 mg, 1.5 mg, 7.5 mg, 15 mg, 75 mg, 150 mg, 300 mg. These doses were administered masked in foods tolerated by the patient that would hide the taste and smell of hazelnut.

In order to standardize the interpretation procedure for each oral challenge test performed, the assessment criteria for the result of each oral challenge test (active agent or placebo) were previously defined based on the signs and symptoms presented during the challenge. The test was considered positive if the following criteria were met: occurrence of objective signs, subjective symptoms with increasing severity assessed in 3 consecutive doses, or persistence of subjective symptoms for over 40 min during the final observation period.

With the aim of assessing the severity of the reaction observed during the controlled exposure test, the following classification was made based on the article by Datema et al. [17]: (a) oropharyngeal symptoms were considered mild; (b) cutaneous, upper respiratory airways and digestive tract symptoms were considered moderate; (c) laryngeal, upper respiratory airways, cardiovascular and neurological symptoms were deemed severe. Anaphylaxis was included in the severe symptom group.

Since the main objective of the study was to identify the association between several variables and the development of severe reactions in the oral challenge test, patients with mild and moderate reactions to hazelnut were included in the same group.

### 2.3. Skin Prick Test

SPT were conducted based on the recommendations of the EAACI [22]. SPT were performed using commercial extracts (Leti SLU, Roxall SA, ALK SA) for tree nuts, pneumoallergens, profilin, polcalcin and Lipid Transfer Protein (LTP). Skin prick by prick tests (SPPT) with hazelnut were also performed.

Tested pneumoallergens included olive, grass, plantain, *Cupressus arizonica* and birch pollens.

Although they belong to different families, testing for other tree nuts (walnuts, almonds, pistachios, cashews) also included peanuts (legumes) and sunflower seeds (seeds) since they are generally eaten mixed with tree nuts.

Histamine hydrochloride (10 mg/mL) was used as positive control and sodium chloride (0.9% NaCl) as negative control. Tests were considered positive when resulting in a papule 3 mm larger than the one caused by the negative control. Skin test results were provided as a mean in mm of the longer and shorter diameters of the papule.

### 2.4. Determination of Specific IgE to Hazelnut

A blood sample was collected from all patients before the challenge test. In each test, part of the supernatant serum was immediately used for the determination of total IgE (ADVIA Centaur^®^ Total IgE, Siemens, Munich, Germany) and sIgE (Immulite^®^, Siemens) to hazelnut whole extract. The remaining supernatant serum was stored at the moment at −40 °C until determination of the levels of sIgE to hazelnut molecular components by means of the ImmunoCAP^®^ techniques (ThermoFisher Scientific, Waltham, MA, USA): rCor a 1, rCor a 8, nCor a 9, rCor a 14, and ALEX^®^ (Macro Array Diagnostics): rCor a 1, rCor a 8, nCor a 9, nCor a 11, rCor a 14.

### 2.5. Statistical Analyses

Absolute (n) and relative (%) frequencies were used to describe qualitative variables, and the mean ± standard deviation (SD) (or median and interquartile range, IQR = Q3 − Q1) were used to express quantitative variables according to their parametric characteristics (Shapiro Wilk normality test).

To analyze the statistically significant differences of the oral food challenges results with respect to baseline patient’s characteristics, skin tests, total and specific IgE, and component-resolved diagnosis results, Chi-square tests (or Fisher’s exact test) were used in the case of qualitative variables. Student’s t-test (normal behavior) or Mann–Whitney U test (non-normal behavior) were applied to compare quantitative variables.

The IBM SPSS statistical software program, v.23 (IBM Corp; Armonk, NY; USA) was used to perform all statistical analyses. P-values below the alpha error (5%) were considered statistically significant.

## 3. Results

### 3.1. Patients Characteristics

Twenty-two patients aged four to 14 were enrolled in the study (mean: 8.1 ± SD: 2.7). 18 boys (81.8%) and four girls (18.2%). The demographical and clinical data of both groups are outlined in Table 1.

No statistically significant differences were found between both groups; thus, it was not possible to associate any demographical or clinical characteristics with the severe reaction group. However, a larger number of patients with AD was found in the group of patients with severe reactions during the challenge test, resulting in a trend, though not statistically significant.

All the patients, except for four in the severe reaction group who avoided all kinds of tree nuts, consumed some nuts other than hazelnut. The most frequently consumed nuts in both groups were almonds and dry sunflower seeds, followed by peanuts and pistachios. The least frequently consumed ones were cashews and nuts.

### 3.2. Food Challenge Results

Of the 22 patients enrolled in the study, three developed a mild reaction in the oral challenge test; 10 had a moderate reaction, and nine presented a severe reaction. For the analysis of the several variables, patients with a mild and moderate reaction were included in a single group (13 patients) to compare them with those developing a severe reaction (nine patients). All patients presented objective symptoms during the hazelnut challenge. Of the total challenges with placebo, only one patient developed oropharyngeal itching at one of the administered doses, which resolved spontaneously within seconds and did not reoccur with subsequent doses.

In 13 (59.1%) of the 22 patients, objective symptoms were preceded by subjective symptoms at one of the previous doses, while in the remaining nine (40.9%) patients, the initial symptoms were directly objective. Subjective symptoms did not meet the criteria described above to consider the test positive, which means that the challenge continued. Most patients (10/13; 76.9%) presenting subjective symptoms before objective ones belonged to the mild/moderate reaction group, while most patients (6/9; 66.7%) who directly presented objective symptoms were in the severe reaction group. These differences were not statistically significant (*p* = 0.079).

In the severe reaction group, the median cumulative hazelnut protein symptom-triggering dose was 99.9 [IQR = 375] mg, while in the mild/moderate reaction group, it was 2.4 [IQR = 97.5] mg. The difference between them was statistically significant (*p* = 0.008).

### 3.3. Skin Prick Tests

Of the 22 patients in the study, nine from the mild/moderate group had a negative prick by prick skin test to hazelnut, and one of them also had a negative prick test to commercial hazelnut extract. No statistically significant differences were observed between the mean size of the papules after the prick or prick by prick test with hazelnut between both groups (Table 2).

However, it was observed that patients in the severe reaction group were more likely to develop a larger papule in the prick test with hazelnut.

No statistically significant differences were found either between both groups in terms of sensitization to pollens or panallergens tested (profilin, polcalcin, LTP). The most common sensitizations were those to grass, olive and *Cupressus arizonica* pollens. Only three patients (one in the mild/moderate group and two in the severe reaction group) did not present pollen sensitization.

**Figure 1:**
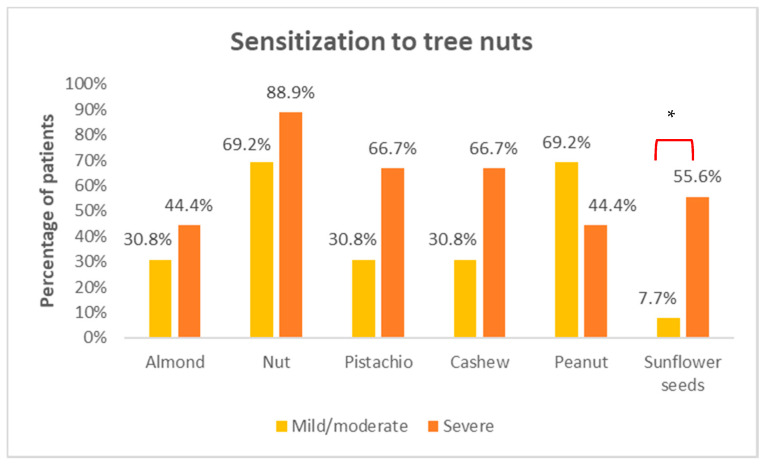
Sensitization to tree nuts. * indicates statistically significant differences (*p* = 0.023) among different groups.

Figure 1 shows the result of the sensitizations found to the different tree nuts. The nuts with the highest sensitization among patients were walnuts (17/22), followed by peanuts (13/22), pistachios (10/22), cashews (10/22), and almonds (8/22). The ones with the lowest sensitization were sunflower seeds, occurring only in six patients of the total. A statistically significant difference was found in terms of sensitization to sunflower seeds, with positivity being much more frequent in the severe reaction group (*p* = 0.023).

### 3.4. Sensitization to Hazelnut Allergens

No statistically significant differences were observed between both groups for the mean total IgE values. The group of patients developing a severe reaction in the hazelnut challenge test presented the highest sIgE values to hazelnut with respect to the mild-moderate group, with this difference being statistically significant (Table 3).

When conducting the hazelnut molecular component analysis with the ImmunoCAP^®^ technique, all the patients presented some level of positivity, except for two patients, one in the mild/moderate group and the other one in the severe reaction group, for whom all the components studied were negative. Most patients (19 out of 22) from both groups presented positivity to one of the storage proteins, nCor a 9 and rCor a 14, with the least represented allergen in the sample being rCor a 1, which was positive in only four patients. rCor a 8 was positive in seven patients. No statistically significant differences were found when comparing positivity of hazelnut molecular components between both groups. However, when studying these components as quantitative variables, higher values of rCor a 14 were observed in the group of patients presenting a severe reaction (Table 3).

Only one patient from the mild/moderate reaction group did not present any sensitization to the hazelnut molecular components analyzed with the ALEX^®^ technique. Most patients (20 out of 22) presented some level of positivity to storage proteins, nCor a 9, nCor a 11, and rCor a 14. The least represented allergen in the sample was rCor a 1, with only three positive patients and rCor a 8 with seven positivities. Cor a 11 was positive in 18 patients, all of the severe reaction group (*n* = 9) and 9 (69.2%) in the mild/moderate reaction group. No statistically significant differences were found when comparing positivity to the hazelnut molecular components between both groups. However, when studying these components as quantitative variables, higher values of nCor a 11 were observed in the group of patients presenting a severe reaction (Table 3).

All qualitative values (positive or negative) of sensitization to the different hazelnut allergens are described in Table S1 of the supplementary material.

## 4. Discussion

In this study, which enrolled 22 pediatric patients allergic to hazelnut in a central region of Spain with no exposure to birch pollen, both the demographic variables and the clinical characteristics were assessed, as well as potential biomarkers (SPT, sIgE, CRD), seeking to establish an association with the development of a severe reaction during the oral challenge test.

### 4.1. Demographic and Clinical Characteristics

Our study did not show any demographic or clinical characteristics associated with the severe reaction group.

A very similar ratio of patients with asthma was found in the two groups. As regards the presence of AD, although it was more prevalent in the severe reaction group, this difference was not statistically significant.

Consistently with our results, other studies did not find an association between severity and the presence of asthma, a history of AD, or the patient’s gender, as in the Petterson et al. study [9]. However, Datema et al. [17] in a sub-study of the EuroPrevall project, which studied 731 subjects (adults and children), found that AD was associated with the severity of the reaction to hazelnut.

The study conducted by Cetinkaya et al. [23], which involved a retrospective study including 184 children allergic to tree nuts, showed that the severity of the reaction was significantly related to the presence of asthma, egg white allergy and female gender. The association between asthma and severity of the reaction is controversial. Thus, the increase in the anaphylaxis risk does not seem to result from the asthma itself, but rather from having uncontrolled asthma [24]. In fact, Summers et al. [15], suggest that what can predict the likelihood of life-threatening acute allergic reactions, rather than the presence or absence of atopic diseases, such as asthma or AD, may be the severity itself of these atopic diseases. Our study did not classify the severity of the patient’s allergic diseases, since that would have entailed creating sub-groups with an excessively small number of patients to analyze, so this potential association was not studied. However, no patient in our study presented uncontrolled asthma, as this was an exclusion criterion for the performance of the oral challenge test. Therefore, this potential risk factor when developing a severe reaction was excluded.

### 4.2. Allergen Dose

A clear difference between both groups was found in our study in terms of the cumulative protein dose triggering a reaction. In the severe reaction group, the cumulative protein dose was clearly higher than in the mild/moderate group. This result is consistent with a potential dose-response curve; at a higher dose, a more severe reaction.

This idea is also reflected in the study conducted by Wainstein et al. [5], who carried out peanut challenge tests in children in which they did not stop the test upon the occurrence of the first subjective or mild objective symptoms, but rather continued administering peanut doses. Most anaphylaxis events occurred after continuing to administer higher amounts of peanut than the ones causing the first reaction.

Another study that is consistent with our findings is the one conducted by Zhu et al. [6] who, after a retrospective analysis of the data published in the literature on the doses at which patients allergic to peanuts developed severe reactions, found that higher doses were associated with more severe reactions compared with doses triggering mild reactions.

On the other hand, there are published data showing an inverse relationship between symptom-triggering doses and reaction severity; the lower the dose, the more severe the reaction. One of these studies is the one conducted by Santos et al. [8] in children allergic to peanuts. In this study, challenges started at higher doses (0.1 g in most patients) than in usual dose range-finding studies, as it was a diagnostic OFC, which the authors suggest allowed them to find a significant correlation between dose and severity. In a different study, mentioned above, Petterson et al. [9] show that the most common reactions usually occur at the lowest doses, although they describe this association as weak, and state that severe reactions also occur at high doses. They propose that this weak association may be due to subject inter-variability due to dose accumulation during the OFC. To avoid this potential confounding factor, Blumchen et al. [13] conducted a modified challenge protocol study, administering food doses every 2 h. However, no association was found between the severity of the reaction and the triggering dose. This result may be explained by what Rolinck-Werninghaus et al. [7] explain in their study, that they do not find any association between the allergen dose and the severity of the reaction, given that all kinds of reactions occur at each of the challenge doses.

Most studies conducted are with peanut or foods other than hazelnut, which means that it may be difficult to establish comparisons between studies. Additionally, the challenge tests performed in our study were proposed as a dose-finding assessment, and thus the initial doses administered were very low (0.15 mg of protein), which entails an important difference from most published studies, where the initial doses are higher. Therefore, it is very important to standardize the challenge test protocol to compare results, since the dose triggering severe reactions in a study that starts at very low challenge doses may seem high, but when compared with the one used in the other study, which began at much higher doses, it may be a low cumulative dose.

Another important factor to consider in our study is that a clear trend was observed in terms of the presence of subjective symptoms before the mild/moderate reaction compared to severe reactions when most patients first presented with objective symptoms. This could indicate that the initial onset of subjective symptoms may reduce the risk of a later severe reaction. These results conflict with those published by Wainstein et al. [5], who describe anaphylaxis not preceded by subjective symptoms as the exception to the rule. In a Europrevall [25] symptom-triggering dose-finding study, it was observed that many patients who presented subjective symptoms later developed objective symptoms when higher doses of the allergen were administered, as happens in our study with mild/moderate reactions, although in the former study, the type of objective reaction the patients presented afterwards is not specified (mild-moderate or severe).

All the results outlined suggest that, as reflected in previous studies, patients probably have two thresholds [5,24], one for a reaction and another one for anaphylaxis. It would be interesting to study the specific characteristics of the different groups based on whether these two thresholds coincide or not and to identify any differences in order to determine which patients will initially have an anaphylactic reaction (reaction threshold = anaphylaxis threshold) since these patients are the ones at higher risk both when performing a controlled challenge test in a hospital setting and of having an accidental reaction in the community.

### 4.3. SPT and Specific IgE to Hazelnut

In our study, we have found a relationship between the levels of specific IgE and the severity of the reaction but not with the SPT or the SPPT. There could be differences in the SPPT due to the amount of allergen in every hazelnut used for the test. Not finding statically differences with the cutaneous tests could be due to the small sample size, since a clear trend is found indicating higher SPT levels in the severe reaction group. Additionally, given the small size of our sample, it was not possible to calculate the necessary diagnostic parameters for sensitivity, specificity, and positive and negative predictive value.

It has been observed that SPT and/or sIgE are predictive of the likelihood of a clinical food reaction, but it remains unclear whether they can predict severity with sufficient discrimination to be of any clinical usefulness. In fact, severe reactions may develop at all levels of sensitization [24].

In the literature, we find both studies that have established this association between severity and level of sensitization to SPT and/or specific IgE to hazelnut [5,8,9,10], as well as other studies that have failed to establish this association [11,12,13,14,15].

In terms of the sensitizations found in the skin tests, walnut was the most frequently observed tree nut, although no statistically significant differences were found in both groups. Finding this sensitization may be secondary to the cross-reactivity between the walnut and hazelnut 2 S albumin storage proteins, as previously described in the literature [26]. This sensitization to walnut found in patients allergic to hazelnut had previously been described in another study in Spanish subjects [27]. However, it should be noted that a statistically significant difference was found in terms of sensitization to sunflower seeds, with positivity being much more frequent in the severe reaction group (five positive results versus one in the mild/moderate reaction group). Although not a tree nut, sunflower seeds are frequently consumed in Spain, often mixed with tree nuts, and it was included in the skin tests for this reason. Of the six patients showing this positivity, the only positive case in the mild/moderate reaction group and in two of the severe reaction group tolerated intake of this seed, which means that it was a sub-clinical sensitization. The 3 remaining cases of the severe reaction group who tested positive for sunflower seeds avoided them, so tolerance could not be confirmed. Several allergens have been identified in sunflower seeds, including an LTP (Hel a 3) and an 2 S albumin (Hel 2 S albumin) [28], one of which could be causing a potential cross-reactivity with the tree nuts. However, further studies should be conducted to identify the true origin of this skin test positivity, as well as the clinical implications entailed for allergic patients developing a severe reaction in our cohort.

### 4.4. Component-Resolved Diagnosis

Sensitization to one hazelnut allergen or another may depend both on the geographical region and on the patient’s age [16]. It is well known that, in countries with high birch pollen exposure (central Europe), there is high sensitization to Cor a 1 (homologous to Bet v1) and that it is usually associated with mild symptoms. However, in Mediterranean countries as the exposure to LTP is high, the predominant sensitization is to Cor a 8 (LTP), and it is often associated with severe reactions [27,29]. So one would expect to find a predominant sensitization to Cor a 8 in our sample and not only 31.8% (*n* = 7). In addition, no patient presented monosensitization to Cor a 8 and most of the patients with positive hazelnut LTP (*n* = 6) were co-sensitized with one of the storage proteins. In Spain, LTP is the allergen most implicated in plant food allergy and specifically, in allergy to Rosaceae fruits, Pru p 3 (peach LTP) is the primary sensitizer [30]. In our area, adolescence is usually the age of onset of peach allergy [31] and the mean age of the patients in our study is younger. This could be one of the causes of having found a lower sensitization to LTP and therefore that the primary sensitizer in hazelnut allergy in our patients has been storage proteins. In fact, the articles in which Cor a 8 is the predominant allergen in hazelnut allergic patients refer to the adult population [32,33,34], which would be in line with this hypothesis.

In children from countries with high birch exposure, it has been observed that primary sensitizations to hazelnut are usually caused by storage proteins (Cor a 9, Cor a 14) [35], and are associated with more severe reactions. In other Southern European countries, it has been observed that children’s allergy to hazelnut is due to sensitization to storage proteins instead of LTP [36,37], as other studies suggest.

In the pediatric population in our study, we can observe also a predominant sensitization to storage proteins (Cor a 9, Cor a 11, Cor a 14), with Cor a 1 and Cor a 8 being the least frequent ones. Cor a 11 sensitization and its implication in hazelnut allergy has not been extensively studied in the literature. In a study by Blanc et al. [34], it is described as a minor allergen recognized only by patients from Mediterranean countries. Considering that in our sample a wide sensitization to this 7 S globulin is observed, being positive in 18 patients (81.8%), more studies should be done to find out its role in hazelnut allergic patients.

In addition to identifying storage proteins as predominant in the sensitization of our patients allergic to hazelnut, a statistically significant difference could be observed, by associating the severity of the reaction with the levels of Cor a 14 (ImmunoCAP) and Cor a 11 (ALEX). The association with hazelnut storage proteins and severe reactions is widely described in the literature, especially for Cor a 9 and Cor a 14 [17,18]. The severity association with Cor a 11 has been described to a lesser extent, since it cannot be included in the diagnostic panels, although there are some published studies, as the one by Verweij et al. [38], which included 40 patients, mostly pediatric, where sensitization to Cor a 11 was predominantly found in children with severe allergies.

## 5. Conclusions

We observed a predominant sensitization to storage proteins in our pediatric patients allergic to hazelnut, in spite of being part of a Mediterranean country with high LTP exposure.

Cor a 11 was one of the most frequents storage proteins found and seems to play an important role in our hazelnut allergic patients.

In our cohort, a statistically significant relationship was found between the severity of the reaction and the highest values of specific IgE to hazelnut, Cor a 11 and Cor a 14, sensitization to sunflower seeds and the symptom-triggering cumulative protein dose.

In addition, a trend was observed with respect to the absence of subjective symptoms prior to severe reactions. This may indicate that subjective symptoms seem to reduce the risk of a later severe reaction.

The most important limitation of our study is the small size of the sample which, among other things, has prevented us from conducting cut-off studies for the biomarkers identified, and other statistical analyses, such as multi-variate studies. So larger studies should be done to confirm these results.

## Figures and Tables

**Table 1:** Demographical and clinical data.

	General (*n* = 22)	Mild/Moderate (*n* = 13)	Severe (*n* = 9)	*p*
Gender				0.264
Male	18 (81.8%)	12 (92.3%)	6 (66. 7%)	
Female	4 (18.2%)	1 (7.7%)	3 (33.3%)	
Age				0.357
mean ± SD	8.1 ± 2.7	8.5 ± 2.6	7.4 ± 2.7	
(min, max)	(4–14)	(5–14)	(4–12)	
Pollen-induced rhinoconjunctivitis				0.380
No	14 (63.6%)	7 (53.8%)	7 (77.8%)	
Yes	8 (36.4%)	6 (46.2%)	2 (22.2%)	
Bronchial asthma				1
No	16 (72.7%)	9 (69.2%)	7 (77.8%)	
Yes	6 (27.3%)	4 (30.8%)	2 (22.2%)	
Atopic dermatitis				0.074
No	8 (36.4%)	7 (53.8%)	1 (11.1%)	
Yes	14 (63.6%)	6 (46.2%)	8 (88.9%)	
Food allergy				0.609
No	5 (22.3%)	2 (15.4%)	3 (33.3%)	
Yes	17 (77.3%)	11 (84.6%)	6 (66.7%)	

**Table 2:** Results of the skin tests with hazelnut.

Skin Tests	Mild/Moderate (*n* = 13)	Severe (*n* = 9)	*p*
Hazelnut SPT			0.077
mean ± SD	5.4 ± 2.3	7.1 ± 1.9	
(min–max)	(0–8)	(5–10)	
Hazelnut SPPT			0.490
mean ± SD	5.8 ± 3.5	6.7 ± 1.9	
(min–max)	(0–13)	(4–9)	

**Table 3:** In vitro diagnosis. Results: ImmunoCAP^®^ in KUA/L. ALEX^®^ in ISU. NA: not available.

		ImmunoCAP^®^ Median [IQR]			ALEX^®^ Median [IQR]	
	Mild/Mod (*n* = 13)	Severe (*n* = 9)	*p*	Mild/Mod (*n* = 13)	Severe (*n* = 9)	*p*
sIgE hazelnut	1.6 [5.1]	14 [37]	*0.042*	NA	NA	
rCor a 1	0 [0.1]	0 [0]	0.683	0 [0]	0 [0.1]	0.691
rCor a 8	0 [1.5]	0.1 [4.1]	0.331	0 [4.7]	0.1 [20.5]	0.498
nCor a 9	0.5 [2.1]	0.9 [11]	0.216	0.4 [1.4]	2.1 [9]	0.225
nCor a 11	NA	NA		0.8 [5.8]	7 [13.2]	*0.049*
rCor a 14	0.3 [0.9]	3.5 [14.9]	*0.027*	9.3 [21.6]	10.5 [36.6]	0.548

## Data Availability

The data presented in this study are available on request from the corresponding author.

## Supplementary Materials

The following are available online at https://www.mdpi.com/article/10.3390/foods10102463/s1, Table S1: Number of patients with positive results to the different in vitro hazelnut allergens.
