# Supplementary material for: Storage Proteins Are Driving Pediatric Hazelnut Allergy in a Lipid Transfer Protein-Rich Area
Source: Foods. 2021 Oct 15;10(10):2463. doi: 10.3390/foods10102463 (PMC8535272; doi:10.3390/foods10102463)
Supplement: Supplementary file 1 [file foods-10-02463-s001.zip › foods-1395601-supplementary.pdf]

Table S1. Number of patients with positive results to the different in vitro hazelnut allergens

In vitro Hazelnut allergens	Total (n=22)	Mild/Moderate (n=13)	Severe (n=9)
Hazelnut sIgE	21	12	9
rCor a 1 (ImmunoCAP®)	4	3	1
rCor a 8 (ImmunoCAP®)	7	4	3
nCor a 9 (ImmunoCAP®)	18	10	8
rCor a 14 (ImmunoCAP®)	18	10	8
r Cor a 1 (ALEX®)	3	2	1
r Cor a 8 (ALEX®)	7	4	3
n Cor a 9 (ALEX®)	13	7	6
n Cor a 11 (ALEX®)	18	9	9
r Cor a 14 (ALEX®)	19	11	8
